# Beneficial Effect of Shikonin on Experimental Colitis Induced by Dextran Sulfate Sodium in Balb/C Mice

**DOI:** 10.1155/2012/271606

**Published:** 2012-12-31

**Authors:** Isabel Andújar, José Luis Ríos, Rosa María Giner, José Miguel Cerdá, María del Carmen Recio

**Affiliations:** ^1^Departamento de Farmacologia, Facultat de Farmàcia, Universitat de València, Burjassot, 46100 Valencia, Spain; ^2^Departamento de Patologia, Facultat de Medicina, Universitat de València, 46100 Valencia, Spain

## Abstract

The naphthoquinone shikonin, a major component of the root of *Lithospermum erythrorhizon*, now is studied as an anti-inflammatory agent in the treatment of ulcerative colitis (UC). Acute UC was induced in Balb/C mice by oral administration of 5% dextran sodium sulfate (DSS). The disease activity index was evaluated, and a histologic study was carried out. Orally administered shikonin reduces induced UC in a dose-dependent manner, preventing the shortening of the colorectum and decreasing weight loss by 5% while improving the appearance of feces and preventing bloody stools. The disease activity index score was much lower in shikonin-treated mice than in the colitic group, as well as the myeloperoxidase activity. The expression of cyclooxygenase-2 was reduced by 75%, activation of NF-**κ**B was reduced by 44%, and that of pSTAT-3 by 47%, as well as TNF-**α**, IL-1**β**, and IL-6 production. Similar results were obtained in primary macrophages culture. This is the first report of shikonin's ability to attenuate acute UC induced by DSS. Shikonin acts by blocking the activation of two major targets: NF-**κ**B and STAT-3, and thus constitutes a promising potential therapeutic agent for the management of the inflammatory bowel disease.

## 1. Introduction

UC is a chronic intestinal disorder with recurrent episodes of remission and relapse [1]. Diffuse mucosal inflammation of the rectum and often a variable length of the colon characterize the disease. The European Crohn's and Colitis Organization (ECCO) differentiates UC into one of three types according to the disease distribution: proctitis (only the rectum is affected), left-sided or distal colitis, and extensive colitis (parts of the colon next to the splenic flexure are also involved) [2]. Patients with UC commonly report symptoms such as diarrhea, rectal bleeding, and fatigue, which adversely impact daily functioning [3]. The incidence of UC differs across countries (0.5 to 24.5 per 100000) and increases being reported in some countries with previously lower incidences, such as those in Eastern Europe and Asia [4]. Although the exact pathogenesis of this disease is poorly understood, infections, environmental factors, complex genetic disorders, and a deregulation of the immune response have been proposed as the main causes of the disease [5–7]. The presence of UC appears to increase the risk of extraintestinal diseases (e.g., iritis/uveitis, primary sclerosing cholangitis, or ankylosing spondylitis) [8], other chronic inflammatory diseases (e.g., arthritis, asthma, or multiple sclerosis) [9], or colorectal cancer [10].

Cytokines are key signals in the intestinal immune system and are known to participate in the disruption of the so-called normal state of controlled inflammation (physiological inflammation of the gut). In inflammatory bowel disease, the innate immune response plays a critical role. Activated dendritic cells and macrophages secrete several cytokines that actively regulate the inflammatory response in UC [11]. Different studies have reported that proinflammatory cytokines such as tumor necrosis factor (TNF)-*α*, interleukin (IL)-1*β*, and IL-6 are increased in the colonic mucosa of UC patients [12]. TNF-*α* expression in human macrophages has been described in the colonic tissue and in macrophages of patients with Crohn's disease and UC. Moreover, serum levels of TNF-*α* correlate with clinical and laboratory indices of intestinal disease activity. IL-1*β* is a proinflammatory cytokine produced predominantly by stimulated macrophages and monocytes and its enhanced production has been detected at both the mRNA and protein levels in patients and in DSS-induced colitis models. In addition, increased productions of inducible nitric oxide synthase and cyclooxygenase-2 have also been found in inflammatory bowel disease, as well as in models of intestinal inflammation [13]. The transcription factor NF-*κ*B is one of the major regulatory components in the complex scenario of inflammatory bowel disease. Obviously the expression and activation of NF-*κ*B is strongly induced in the inflamed gut of inflammatory bowel disease patients. NF-*κ*B is not just expressed but is also in a state of activation in mucosal macrophages and epithelial cells in inflammatory bowel disease patients. The amount of activated NF-*κ*B correlates with the severity of intestinal inflammation. The increased NF-*κ*B expression in macrophages is accompanied by an increased capacity of these cells to produce and secrete TNF-*α*, IL-1*β*, and IL-6, but predominantly NF-*κ*B-induced cytokines are responsible for further stimulation, activation, and differentiation of *lamina propria* immune cells, resulting in the perpetuation of mucosal inflammation [14]. In fact, many of the immunosuppressive drugs currently used in inflammatory bowel disease treatment, such as corticosteroids, sulfasalazine, methotrexate, and anti-TNF-*α* antibodies, are known to mediate their anti-inflammatory effects, at least partly, via inhibition of this transcription factor.

The goal of treatment for inflammatory bowel disease is to eliminate inflammation in order to provide symptom relief and mucosal healing. Aminosalicylates remain the cornerstone of therapy for patients with active, mild-to-moderate UC. However, its role in Crohn's disease has been more controversial. Corticosteroids also remain popular medications for inducing remission in both UC and Crohn's disease. However, they cannot effectively maintain remission, and their significant adverse effects limit their long-term use. When inflammatory bowel disease patients do not respond to first-line therapy, the immunosuppressive therapy with azathioprine and 6-mercaptopurine is often used. However, only 40% of patients receiving azathioprine remain in remission after one year. Thus, additional safe and effective treatment options are needed for these patients [15].

Despite the advancements that have been made in treating patients with anti-TNF therapy and other drugs, a large number of patients still need surgery. 

Because of a lack of safety and efficacy of standard therapies, the use of complementary therapies such as natural products and dietary components is fast becoming an attractive approach for the treatment of inflammatory bowel disease. Several plant constituents have been reported to be useful in the management of inflammatory bowel disease and other chronic inflammatory processes [16]. Among these, polyphenols such as rutin [15], thearubigin [17], or curcumin [18, 19] and other polyphenols exhibit intestinal anti-inflammatory activity [20]. The activity of these natural molecules can be associated with broad inflammatory parameters including morphological parameters, myeloperoxidase activity, glutathione levels, cytokine production (TNF-*α*, IL-1*β*, and IL-6), p38-MAPK, and NF-*κ*B activation.

**Figure 1:**
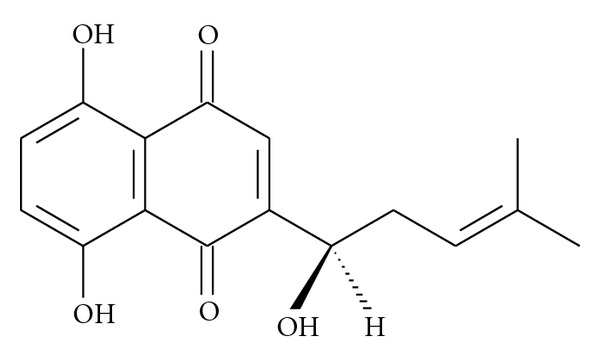
Chemical structure of shikonin.

Traditional Chinese medicine continues to provide front-line pharmacotherapy for many millions of people worldwide. One example is the root of *Lithospermum erythrorhizon* Sieb. et Zucc. (Boraginaceae) applied to treat macular eruptions, measles, sore throats, carbuncles, and burns [21]. A major component of this plant is shikonin (Figure 1), a natural naphthoquinone derivative. This compound has been reported to possess different medicinal properties such as antibacterial, wound healing, anti-inflammatory, antithrombotic, and antitumor effects [22]. In a previous work, our group has demonstrated that the dichloromethane extract of *L. erythrorhizon* inhibited acute ear and paw edema induced in mice via topical application of TPA and plantar subcutaneous injection of carrageenan, respectively [23]. We also demonstrated that the main compound present in this extract, shikonin, reduces 12-*O*-tetradecanoylphorbol 13-acetate-induced acute ear inflammation *in vivo* by inhibiting NF-*κ*B translocation to the nucleus as well as by interfering with I*κ*B*α* degradation. Both of these effects were also observed *in vitro* in the murine RAW 264.7 macrophage cell line [24]. In the present study, we examined the effects of shikonin on changes in clinical symptoms and expression of various proinflammatory mediators in DSS-induced colitis mice. This experimental model is widely used to study the pathogenic mechanisms underlying inflammatory bowel disease as well as to evaluate new intestinal anti-inflammatory agents. Because this murine model resembles human UC, it allows us to evaluate whether shikonin could serve as a new therapeutic tool for treating patients suffering from this disease.

## 2. Materials and Methods

### 2.1. Chemicals

Shikonin was purchased from TCI Europe (Zwijndrecht, Antwerp, Belgium). All chemical and biochemical reagents were purchased from Fluka Chemika-Biochemika (Buchs, Switzerland), Baker (Deventer, Holland), Panreac (Barcelona, Spain), and Sigma-Aldrich (St. Louis, MO, USA).

### 2.2. Animals

Female Balb/C mice weighing 18–20 g (Harlan Interfauna Ibérica, Barcelona, Spain) were used for *in vivo* experiments. All animals were fed a standard diet *ad libitum* and housed under a 12 h light/dark cycle at 22°C and 60% humidity. Housing conditions and all *in vivo* experiments were approved by the Institutional Ethics Committee of the University of Valencia, Spain, according to the normative of October 2005 (RD1201/2005).

### 2.3. Induction of Acute UC and Treatment with Shikonin

Acute colitis was induced by oral administration of 5% DSS (molecular weight 36–50 kD; MP Biomedicals LLC, Solon, OH, USA) (w/v) in fresh tap water *ad libitum* for seven days (6–10 mice per group). Three other groups of animals which also received 5% DSS in drinking water were treated orally with shikonin twice during the experiment (on day 1 and on day 5) at different doses (6.25, 12.5, and 25 mg kg^−1^). Shikonin was dissolved in water : ethanol : Tween 80 (10 : 1 : 1) and administered orally with the aid of a gavage. A fifth group received only fresh tap water (Figure 10). Water consumption was controlled for all groups, and no major differences were detected. The disease activity index was determined according to the parameters outlined in Table 1 [25]. On termination of the experiment on day eight, mice were killed by cervical dislocation, and colonic tissues were dissected for further analysis. This same protocol was carried out three times in independent experiments.

### 2.4. Histology

Small (approximately 1 cm) sections of excised colonic tissue were fixed in 10% paraformaldehyde in phosphate-buffered saline (PBS, pH 7.4) and embedded in paraffin. Sections (4 *μ*m) were cut and stained with haematoxylin and eosin. Histologic assessment of colonic mucosa was carried out in a blinded fashion by a pathologist as described previously (Table 2) [26].

### 2.5. Immunofluorescence Staining

The frozen sections of the distal colon were fixed in in freshly prepared 4% paraformaldehyde in PBS buffer (pH 7.2) and stained to detect lymphocyte and macrophage infiltration by immunofluorescence using fluorescein isothiocyanate-(FITC-)conjugated rat anti-mouse CD4+ monoclonal antibody (Miltenyi Biotec, Gladbach, Germany) or allophycocyanin-(APC-)conjugated rat anti-mouse CD11b+ monoclonal antibody (Miltenyi Biotec), respectively. The sections were mounted with Fluoroshield mounting medium with 4′,6′-diamidino-2-phenylindole (DAPI) (Sigma-Aldrich). The numbers of CD4+ and CD11b+-positive areas were measured on five randomly chosen visual fields at 200× magnification.

### 2.6. Myeloperoxidase Activity Assay

Colon samples from mid to distal colon were rinsed with cold PBS, blotted dry, and immediately frozen with liquid nitrogen. The samples were stored at −80°C until use. For myeloperoxidase activity determination, samples were ground to powder in a mortar. Myeloperoxidase activity was determined as described by Suzuki et al. [27]. Briefly, about 40 mg of tissue was weighed for each sample and homogenized in 80 mM monopotassium phosphate/disodium phosphate buffer (pH 5.4) containing 0.5% hexadecyltrimethylammonium bromide. After centrifugation, 100 *μ*L of PBS, 85 *μ*L of 22 mM monopotassium phosphate/disodium phosphate buffer, and 15 *μ*L of hydrogen peroxide 0.017% were added to 30 *μ*L of the supernatant. The enzymatic reaction begins when adding 20 *μ*L of tetramethylbenzidine hydrochloride. After 3 min at 37°C the reaction is stopped. Absorbance is read at 630 nm. Myeloperoxidase activity is expressed as the amount of enzyme required to convert 1 *μ*mol of hydrogen peroxide to water in 1 min, expressed per gram of wet weight of tissue.

### 2.7. Preparation of Cytosolic and Nuclear Fractions from Colon

Protein extraction from the intestine was performed as described previously by Sugimoto et al. [18]. In brief, tissues were homogenized for 1 min with a Polytron PT-2000 (Kinematica AG, Lucerne, Switzerland) tissue homogenizer in 1.5 mL of ice-cold buffer A (10 mM 4-(2-hydroxyethyl)-1-piperazineethane sulfonic acid (HEPES) pH 7.9, 10 mM KCl, 1.5 mM MgCl_2_, 0.5 mM dithiothreitol, 0.1 mM ethylenediaminetetraacetic acid (EDTA), 0.5 mM phenylmethyl sulphonyl fluoride, 1 *μ*g mL^−1^ aprotinin, 1 *μ*g mL^−1^ leupeptin, and *μ*g mL^−1^ pepstatin A). Igepal CA-630 was added to a final concentration of 0.5%. The homogenates were chilled on ice with gentle shaking for 45 min. The membrane fraction was precipitated by means of centrifugation at 106 ×g for 10 min at 4°C. The supernatant containing the cytosolic fraction was stored at −80°C until use. The pellet was resuspended with vortex in 500 *μ*L of buffer B (20 mM HEPES pH 7.8, 400 mM NaCl, 1.5 mM MgCl_2_, 0.2 mM EDTA, 25% glycerol, 0.5 mM phenylmethylsulphonyl fluoride, 0.5 mM dithiothreitol, 1 *μ*g mL^−1^ aprotinin, 1 *μ*g mL^−1^ leupeptin, and 1 *μ*g mL^−1^ pepstatin A) and chilled during 30min on ice with gentle shaking. After centrifugation at 20800 ×g for 15 min at 4°C, the supernatant containing the nuclear fraction was removed and stored at −80°C until use. Cell lysates (30 *μ*g of protein) were boiled in sodium docecyl sulphate sample buffer for 5 min before undergoing electrophoresis. 

### 2.8. Western Blot Analysis for Cyclooxygenase-2, NF-*κ*B p65, and Phosphorylated Signal Transducer and Activator of Transcription (pSTAT)3

After extraction, the presence of proteins in the supernatants was determined by means of the Bradford method with bovine serum albumin as the standard. Equal amounts of protein (30 *μ*g) were then loaded onto 10% sodium dodecyl sulphate polyacrylamide electrophoresis gel and transferred onto polyvinylidene difluoride membranes at 125 mA for 90 min. The membranes were then blocked in PBS-Tween 20 containing 3% w/v defatted milk. For cyclooxygenase-2, the membranes were incubated with anticyclooxygenase-2 polyclonal antibody (1 : 1000), obtained from Cayman (Ann Arbor, MI, USA). For p65, the membranes were incubated with anti-p65 polyclonal antibody (1 : 500 dilution, SC-7151); for pSTAT-3, the membranes were incubated with anti-pSTAT-3 (SC-8019, 1 : 500 dilution); and for poly(ADP-ribose) polymerase (PARP), the membranes were incubated with anti-PARP polyclonal antibody (SC-1562, 1 : 400 dilution), all of them purchased from Santa Cruz Biotechnologies (Santa Cruz, CA, USA). Finally, for *β*-actin, the membranes were incubated with anti-*β*-actin polyclonal antibody (1 : 10000 dilution), obtained from Sigma-Aldrich. The blots were washed and incubated with peroxidase-conjugate anti-rabbit, anti-mouse, or anti-goat immunoglobulin G (1 : 12000 dilution; Cayman). The immunoreactive bands were visualized with the aid of an enhanced chemilumeniscence system (Millipore Corporation, Billerica, MA, USA).

### 2.9. Culture of Colon Organ Cells

After the *in vivo* protocol, the colon of three mice was removed, opened longitudinally and washed in PBS, as previously described by Siegmund et al. [28]. The colon was then further cut into 1 cm segments and cultured in complete Roswell Park Memorial Institute (RPMI)-1640 medium supplemented with 10% fetal calf serum, penicillin (100 U mL^−1^), and streptomycin (100 U mL^−1^) in a 24-well plate. After 24 h, supernatant was collected and stored at −80°C until use and each intestinal segment was weighed.

### 2.10. Mouse Peritoneal Macrophages Isolation

Balb/C mice from a separate set of experiments were given a 1.5 mL intraperitoneal injection of 3% thioglycollate in water. Four days later, mouse peritoneal macrophages were collected by peritoneal lavage with PBS, pelleted, and washed in PBS. Cells were plated at a density of 1 × 10^6^ cells mL^−1^ with Dulbecco's Modified Eagle's Medium (DMEM) supplemented with 10% fetal calf serum, penicillin (100 U mL^−1^), and streptomycin (100 U mL^−1^). Cells were given 2 h to adhere, medium was changed, and adherent cells were used for cytokine and nitrite assays.

### 2.11. RNA Extraction and Reverse Transcription-Polymerase Chain Reaction (RT-PCR)

Mouse peritoneal macrophages (10^6^ cells per well) were pretreated with shikonin for 1 h and then stimulated with lipopolysaccharide (1 *μ*g mL^−1^) for 3 h. Cells were collected, and total RNA was extracted with the aid of RNeasy mini spin (50) columns (Qiagen, Hilden, Germany), in accordance with the manufacturer's instructions. The concentration of the extracted RNA was calculated by measuring the optical density at 260 nm. The ratio of the optical density at 260 nm to that at 280 nm was always higher than 1.8. Aliquots of 1 *μ*g of RNA were transformed to first strand complementary deoxyribonucleic acid (cDNA) with AffinityScript Multiple Temperature Reverse Transcriptase (Agilent Technologies, Santa Clara, CA, USA). Then, 1 *μ*L of the resulting cDNA was mixed with 0.75 *μ*M primers (Invitrogen, Langley, OK) of IL-6 (sense: 5′-ATGCTGGTGACAACCACGGCC-3′; antisense: 5′-GGCATAACGCACTAGGTTTGCCGA-3′), IL-1*β* (sense: 5′-GCTGGAGAGTGTGGATCCCAAGCA-3′; antisense: 5′-AGCGACCTGTCTTGGCCGAGG-3′), TNF-*α* (sense: 5′-AGCCCACGTCGTAGCAAACCAC-3′; antisense: 5′-TAGACCTGCCCGGACTCCGC-3′). *β*-Actin primers were purchased from R&D Systems (Minneapolis, MN, USA). The thermocycler conditions were 94°C for 1min, with an annealing temperature of 55°C for 1 min and an elongation temperature of 72°C for 1 min for the first 30 cycles, followed by an elongation temperature of 72°C for 10 min. After the reaction was completed, the amplified product was removed from the tubes and run on 1% agarose gel.

### 2.12. Measurement of Cytokines

The concentrations of various cytokines in the culture supernatants of the colons and of mouse peritoneal macrophages were measured using an enzyme-linked immunosorbent assay kit (eBioscience, San Diego, CA, USA) according to the manufacturer's instructions.

### 2.13. Nitrite Determination by Griess Assay

Nitric oxide levels were assessed by nitrite quantification as described elsewhere [29]. Briefly, 100 *μ*L of mouse peritoneal macrophages culture medium was incubated for 15 min with Griess reagent (Sigma-Aldrich). Absorbance was read at 540 nm.

### 2.14. Software

Images for all western blot and PCR experiments were acquired with the image analysis system LAS-3000 mini (Fujifilm, Tokyo, Japan). Digital images were processed and band density measurements were made with the aid of a Multi Gauge V3.0 software package (Fujifilm).

### 2.15. Statistical Analysis

Statistical analysis was performed by means of one-way analysis of variance (ANOVA) and Dunnett's *t*-test. The results are presented as the mean ± SEM GraphPad Prism 4.0 software (GraphPad Software Inc., San Diego, CA, USA) was used for all calculations.

## 3. Results and Discussion

### 3.1. Effects of Shikonin Treatment on Clinical Symptoms

We assessed the systemic effect of shikonin using the acute DSS-induced UC model. For seven days mice received either fresh tap water or 5% DSS dissolved in fresh tap water. Three other groups received 5% DSS and were administered shikonin (6.25, 12.5, or 25 mg kg^−1^) orally both at the beginning of the experiment and on day five (*n* = 6–10 for each group). 

**Figure 2:**
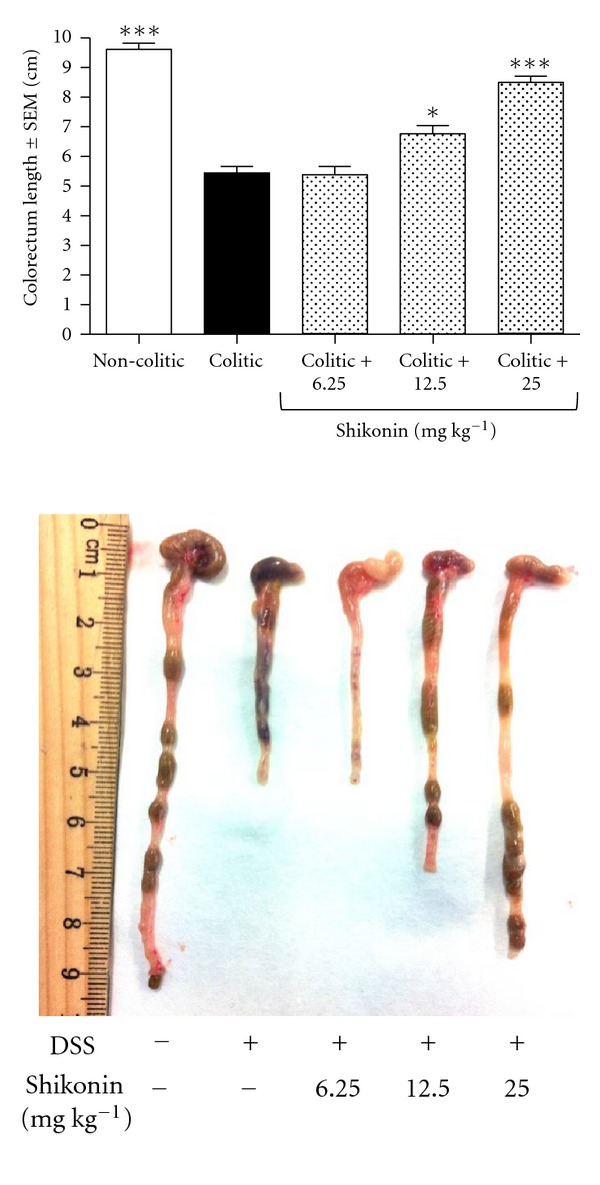
Effect of shikonin on colon length. The noncolitic group received fresh tap water *ad libitum*, and the colitic group received fresh tap water with 5% DSS added to the water during 7 days. Three other groups of mice received the same tap water with 5% DSS and were treated with two doses of 6.25, 12.5, or 25 mg kg^−1^ of shikonin on days 1 and 5 of the experiment. At the end of the experiment, all mice were killed by cervical dislocation and large intestines were removed. After washing with ice-cold phosphate-buffered saline, they were placed on filter papers and measured without the cecum. Statistical analysis was performed using one-way analysis of variance followed by Dunnett's *t*-test. *P < 0.05, ***P < 0.001 versus colitic group. The results shown are representative of three independent experiments with 6 to 10 mice per group.

**Figure 3:**
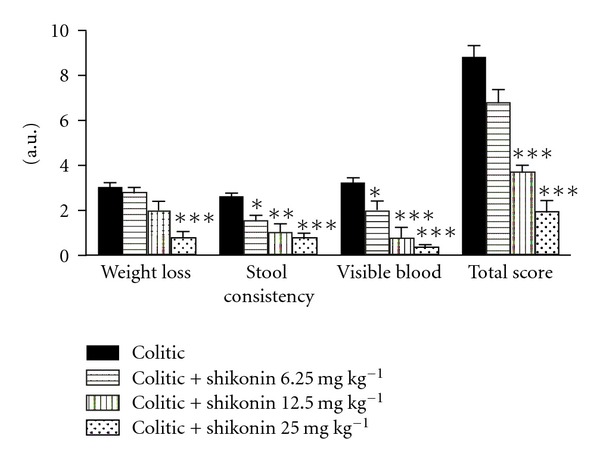
Effect of shikonin on the disease activity index. Disease activity index was evaluated for colitic groups (black bars) and shikonin-treated colitic groups on termination of the experiment according to the scoring on Table 1. ***Significantly different from the colitic group (*P* < 0.001; one-way analysis of variance followed by Dunnett's *t*-test).

The DSS-induced murine UC model used in this study is a well-established general prototype of intestinal tissue damage that accurately resembles the histological aspects of the tissue damage observed in patients suffering from inflammatory bowel disease [30]. In this experimental protocol of acute colitis, shikonin was found to exert an anti-inflammatory effect after only two administrations (Figure 2) in a dose-dependent manner. In the case of the colorectum length, colitic mice suffered a shortening of 43% when compared to noncolitic mice. Shikonin significantly prevented this shortening being only 30% at a dose of 12.5 mg kg^−1^ (6.77 ± 0.22 cm versus 9.61 ± 0.20 cm in noncolitic mice, *P* < 0.05) and 11% at a dose of 25 mg kg^−1^ (8.51 ± 0.19 mm versus 9.61 ± 0.20 cm in noncolitic mice, *P* < 0.001). Moreover, shikonin-treated mice showed improvements in body weight, appearance of feces and alleviation of bloody stool. The disease activity index score, an indicator of the severity of intestinal inflammation, was 8.79 ± 0.53 in the colitic group. Shikonin treatment resulted in a significantly lower disease activity index score, being 3.75 ± 0.25 in the group treated with shikonin 12.5 mg kg^−1^ and 1.90 ± 0.53 in the group treated with shikonin 25 mg kg^−1^ (Figure 3). Shikonin thus ameliorated all symptoms of DSS-induced inflammation in mice (*P* < 0.0001). 

Since the best macroscopic results were obtained with the dose of 25 mg kg^−1^ of shikonin, we continued the rest of the experiments with the samples obtained from this group.

**Figure 4:**
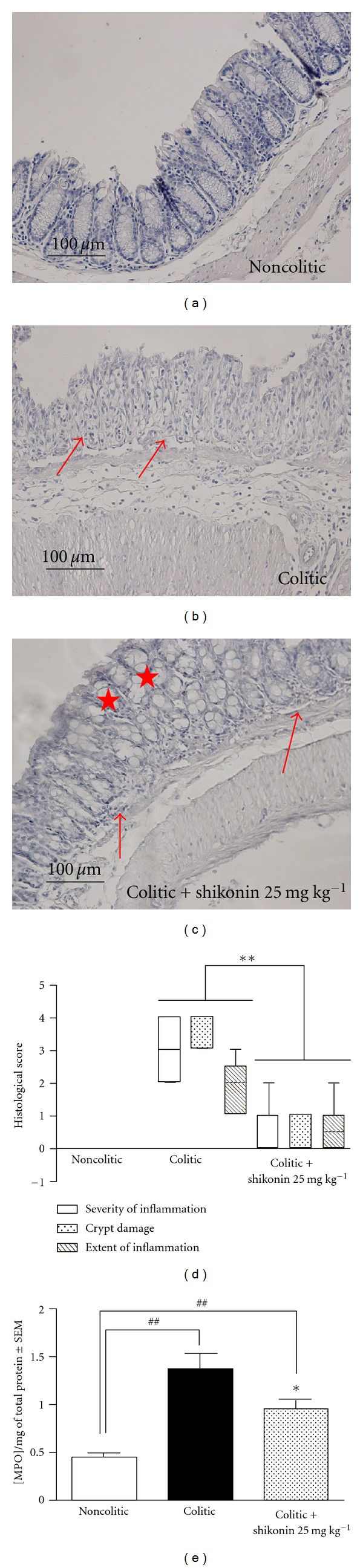
Effect of shikonin treatment on histological parameters in acute ulcerative colitis. Three representative colonic haematoxylin/eosin sections: mice received either fresh tap water (a), fresh tap water with 5% DSS (b), or fresh tap water with DSS 5% and treatment with shikonin 25 mg kg^−1^ (c) as described in Section 2. Inflammatory infiltrate (arrow); replenishment of goblet cells with mucin (star). (d) The severity of inflammation (white bars), extent of inflammation (dotted bars), and crypt damage (lined bars) were determined as described in Table 2. The data presented are representative of three independent experiments with 6 to 10 mice per group. The boundary of the box indicates the 25th and 75th percentiles; the line within the box marks the median. Whiskers indicate the 90th and 10th percentile. **Significantly different from the total histological score of the colitic group, determined by means of a one-way analysis of variance followed by Dunnett's *t*-test (*P* < 0.01). (e) Mucosal myeloperoxidase levels were measured to evaluate the effect of shikonin on the number of neutrophils infiltrating the colon. *P < 0.05, **P < 0.01; significantly different from the colitic group; ^#^ *P* < 0.05, ^##^ *P* < 0.01; significantly different from the noncolitic group, determined by means of a one-way analysis of variance followed by Dunnett's *t*-test (*n* = 6).

Histological analyses revealed that shikonin 25 mg kg^−1^ treatment improved colonic architecture in comparison with that of colitic mice; the treated group was assigned a tissue damage score of 1.67 ± 0.34 versus 8.47 ± 0.37 for the colitic group (*P* < 0.001). Colitic mice exhibited marked cryptic distortion, erosion of the *lamina propria* mucosae, inflammatory cell infiltration, and the disappearance of mucus membrane cells (Figure 4(b)). Treatment with shikonin actually restored the damaged colon, which displayed a moderate inflammatory infiltrate (arrow). Restoration of crypt architecture with goblet cell replenishment of mucin was also observed (star) (Figure 4(c)). 

**Figure 5:**
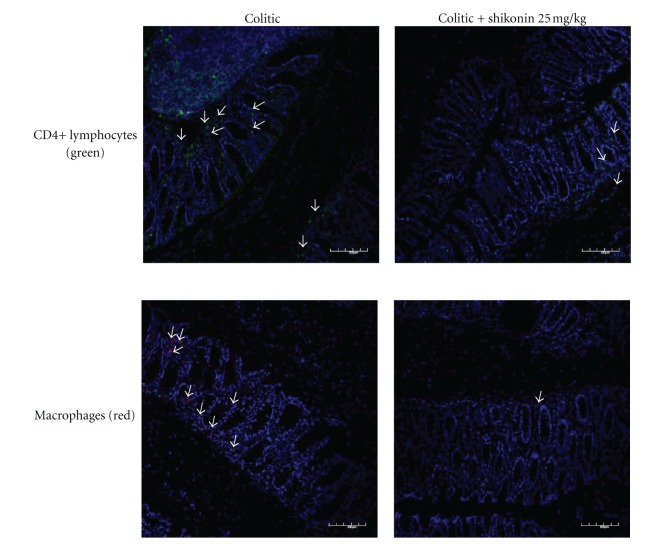
Immunofluorescence detection of CD4+ lymphocytes and CD11b+ cells. Shikonin treatment reduces the presence of CD4+ lymphocytes and macrophages in the colon tissue of mice. Colons were obtained from the colitic group and from the shikonin-(25 mg kg^−1^) treated group and were processed for immunofluorescence analysis using CD4+ FITC-conjugated monoclonal antibody and CD11b+ APC-conjugated monoclonal antibody; representative results from four independent animals are shown. Arrows indicate the positive stained cells. Original magnification, 200×.

By immunofluorescence, we observed a large number of lymphocytes and macrophages in the colonic samples from colitic mice, mainly located in the mucosa. Shikonin treatment prevented this infiltration (Figure 5).

Consistent with these histologic and immunofluorescence findings, we can observe in Figure 4(e) an increase in colonic myeloperoxidase activity in colitic animals. Oral administration of 25 mg kg^−1^ of shikonin is able to significantly reduce in a 38% (*P* < 0.05) of this increase.

Taken together, our results indicate that shikonin can markedly reduce inflammatory cell infiltration in the colon of colitic mice.

### 3.2. Shikonin Limits Colonic Proinflammatory Cytokine Expression

**Figure 6:**
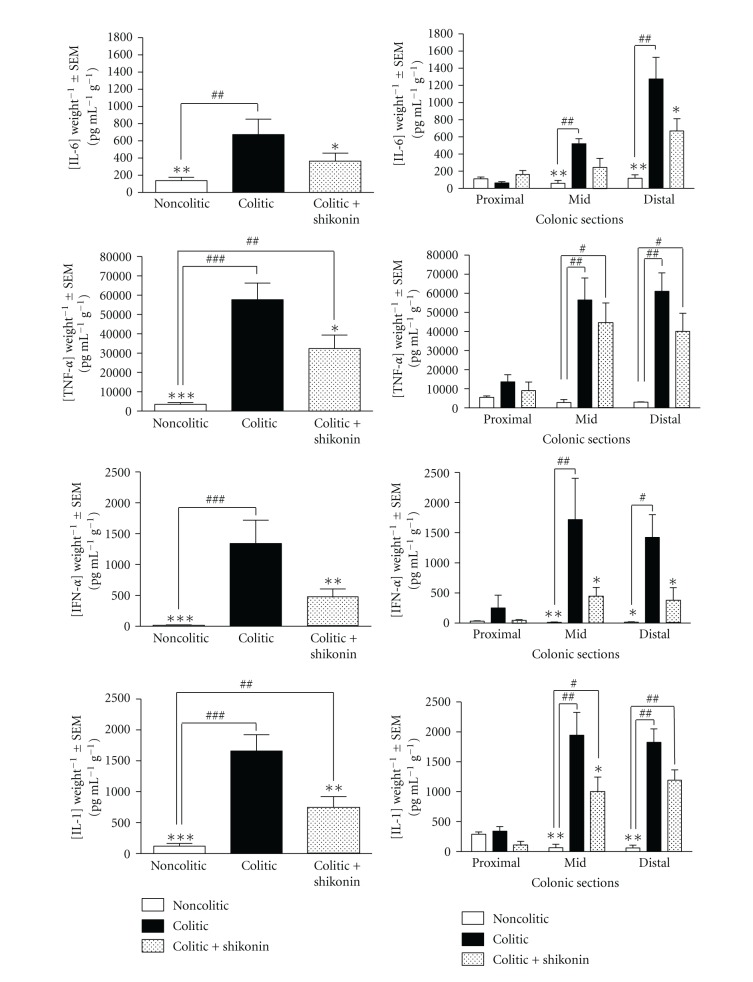
Expression of cytokines in the supernatant of colonic culture by ELISA. Colons were cultured for 24 h, supernatants were collected, and cytokines were measured in duplicate with ELISA. Left panels represent the levels of cytokines measured in the whole colon (after removing cecum and anus); right panels represent the levels of cytokines measured in the different areas of the colon (proximal, mid, and distal area). Results are expressed as the concentration of cytokine referred to wet weight (pg mL^−1 ^mg^−1^) ±SEM and are representative of three independent experiments with 2 colons per group. Differences with the colitic group were determined by means of a one-way analysis of variance followed by Dunnett's *t*-test (*P < 0.05, **P < 0.01; significantly different from the colitic group; ^#^ *P* < 0.05, ^##^ *P* < 0.01; significantly different from the noncolitic group).

The recruitment of circulating leukocytes into the colon leads to the release of proinflammatory mediators, including proinflammatory cytokines such as IL-1*β*, IL-6, IFN-*γ*, and TNF-*α* [31] and proinflammatory synthases such as inducible nitric oxide synthase and cyclooxygenase-2 [32, 33]. To determine whether shikonin limits the production of proinflammatory cytokines, therefore protecting the colonic mucosa, we measured the levels of IL-6, TNF-*α*, IFN-*γ*, and IL-1*β* in the supernatant of cultured colons of 5% DSS-exposed mice given control diet or 25 mg kg^−1^ of shikonin. As Figure 6 shows, treatment with shikonin significantly reduces the levels of these proinflammatory cytokines in about 50% when determined in the whole colon. Since DSS-induced UC in mice appears to be more severe in the distal colon, we measured the production of cytokines in each area of the colon and analyzed the effect of shikonin per area. As shown in this same Figure, DSS significantly increases the production of all proinflammatory cytokines both in mid and in distal colon. Shikonin tends to decrease the production of all cytokines analyzed in both areas. In the mid colon, treatment with shikonin results in a 61% reduction of the production of IL-6, 29% reduction of TNF-*α*, 75% reduction of IFN-*γ*, and 51% reduction of IL-1*β*, although these inhibitions were statistically significant only for IFN-*γ* and IL-1*β*. In the distal colon, as in the case of the mid colon, the production of all measured cytokines was reduced, being the inhibition of IL-6 (52%) and IFN-*γ* (74%) statistically significant.

These data suggest that shikonin may lessen the damaging effects of DSS by suppressing the expression of proinflammatory cytokines. These results agree with Dai et al. (2009) [32], who used a model of collagen-induced arthritis to show that the antiarthritic activity of shikonin occurs through inhibition of Th1 cytokines.

### 3.3. Effect of Shikonin on Nitric Oxide Production, Cytokine Production, and Gene Expression in Mouse Peritoneal Macrophages

**Figure 7:**
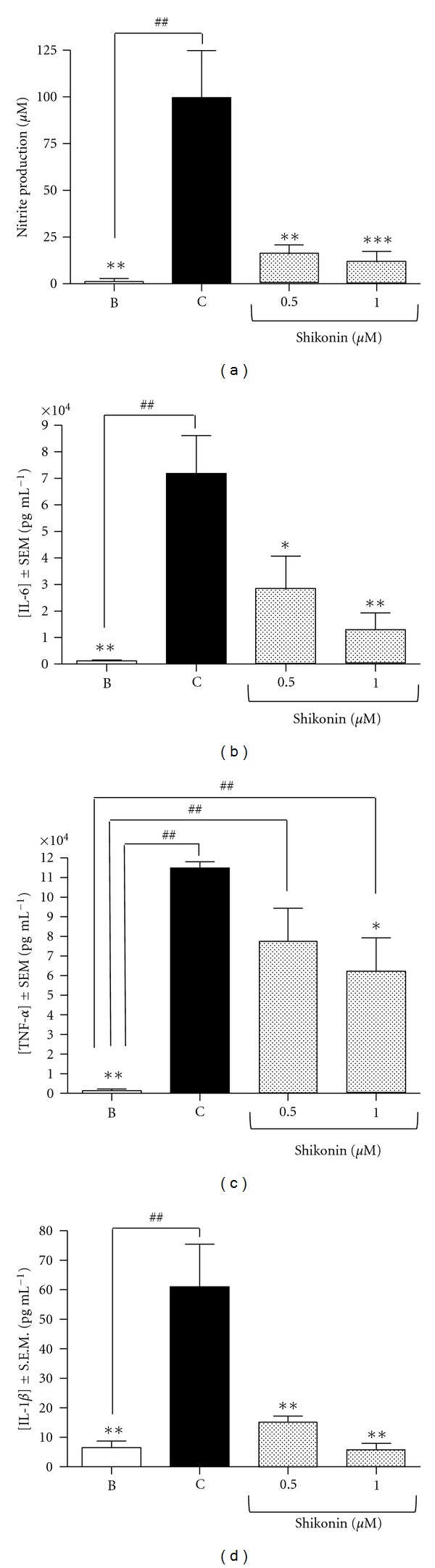
Effect of shikonin on nitric oxide and overall cytokine production in peritoneal macrophages isolated from Balb/C mice. Mouse peritoneal macrophages were isolated and cultured for 24 h. Supernatants were collected, and nitrites and cytokines were measured in duplicate. The B (blank) group represents untreated and unstimulated macrophages, C (control) group represents those macrophages stimulated with lipopolysaccharide (1 *μ*g mL^−1^), and finally two other groups were treated for 1 h with different doses of shikonin (0.5 and 1 *μ*M) prior to stimulation with lipopolysaccharide. Results are representative of ten independent experiments. Differences were determined by means of a one-way analysis of variance followed by Dunnett's *t*-test (*P < 0.05, **P < 0.01, ***P < 0.001 significantly different from the control group; ^##^ *P* < 0.01, significantly different from the blank group).

To help explain the amelioration in the curse of the disease observed in colitic mice treated with shikonin, we studied *in vitro* the effect of this naphthoquinone on the production of nitric oxide and proinflammatory cytokines in lipopolysaccharide-stimulated peritoneal macrophages from female Balb/C mice. As shown in Figure 7(a), shikonin 0.5 *μ*M counteracts significantly the production of nitric oxide (85%).

Moreover, the release of IL-6 (62%) and IL-1*β* (84%) is also significantly reduced at 0.5 *μ*M and at 1 *μ*M shikonin inhibits significantly the release of TNF-*α* (46%) (Figures 7(b)–7(d)).

**Figure 8:**
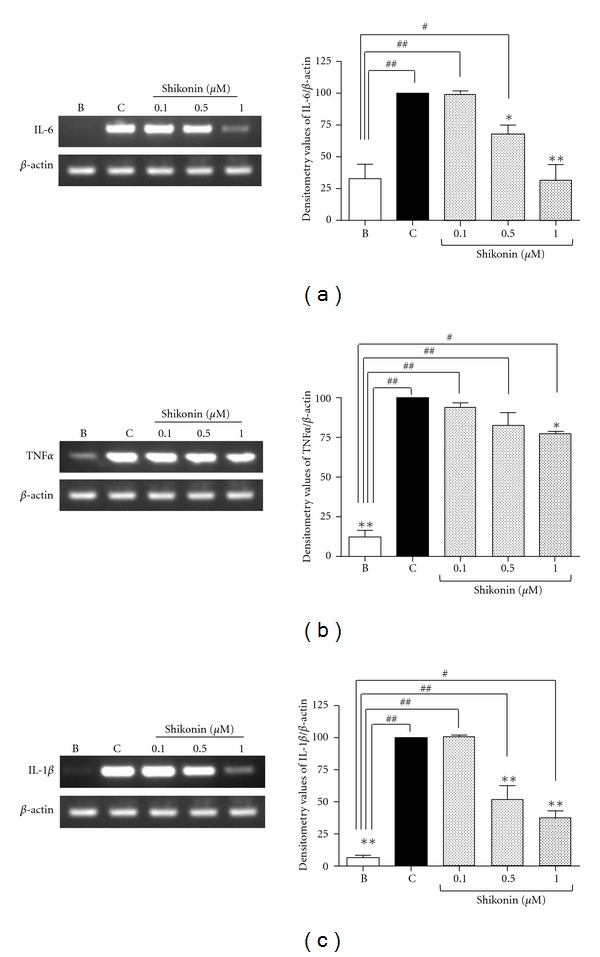
Expression of proinflammatory genes in peritoneal macrophages isolated from Balb/C mice. Proinflammatory gene expression levels were measured using RT-PCR, as described in Section 2. Representative photographs from ten independent experiments with each gene are shown. *β*-Actin served as the internal control. The expression seen in the lipopolysaccharide-stimulated group (control) was standardized as 100% expression. *P < 0.05, **P < 0.01; significantly different from the control group; ^#^ *P* < 0.05, ^##^ *P* < 0.01; significantly different from the blank (unstimulated) group, determined by means of a one-way analysis of variance followed by Dunnett's *t*-test (*n* = 10).

To provide further insight into the mechanism underlying the anti-inflammatory activity demonstrated by shikonin, mRNA expression levels in peritoneal macrophages of these same proinflammatory cytokines were measured by RT-PCR. As shown in Figure 8, mRNA levels for IL-6, TNF-*α*, and IL-1*β* in lipopolysaccharide-stimulated macrophages were significantly increased. Those increases were significantly attenuated in shikonin pretreated macrophages in concentrations of 0.5 and 1 *μ*M. At 0.5 *μ*M shikonin significantly inhibits the expression of IL-6 (37%, *P* < 0.05) and IL-1*β* (32%, *P* < 0.01); at 1 *μ*M shikonin significantly inhibits the expression of IL-6 (85%, *P* < 0.01), TNF-*α* (20%, *P* < 0.05), and IL-1*β* (87%, *P* < 0.01).

### 3.4. Shikonin Decreases Cyclooxygenase-2 Protein Expression and Nuclear Translocation of NF-*κ*B p65 and pSTAT-3

**Figure 9:**
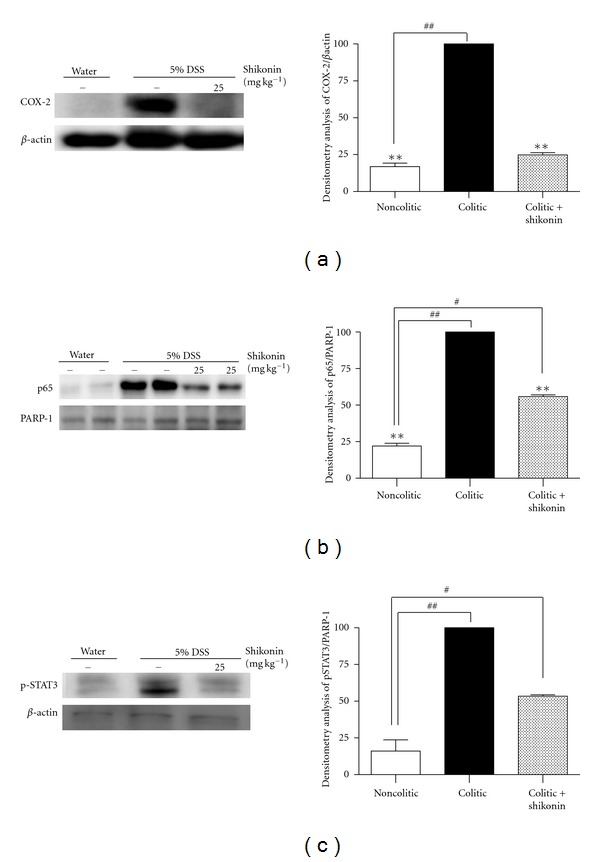
Effects of shikonin on cyclooxygenase-2 expression and on nuclear translocation of p65 and phosphorylated signal transducer and activator of transcription 3. The left panels show an example of western blot following probing with the corresponding antibody. The histograms at the right represent the data derived from the western blots following densitometry analysis. Levels were normalized against *β*-actin or PARP-1 antibody. **P < 0.01 significantly different from the colitic group; ^#^ *P* < 0.05, ^##^ *P* < 0.01 significantly different from the noncolitic group, determined by means of a one-way analysis of variance followed by Dunnett's *t*-test (*n* = 6–10).

**Figure 10:**
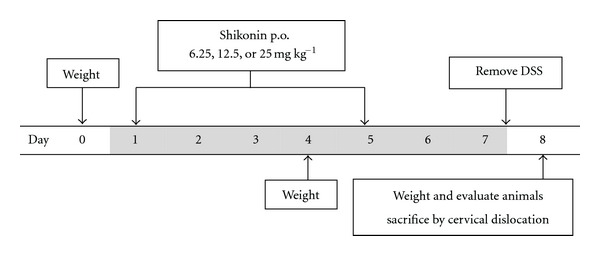
Induction of acute ulcerative colitis and treatment protocol.

COX-2 plays an important proinflammatory role in the pathogenesis of inflammatory bowel disease. We thus evaluated the effect of shikonin on colonic cyclooxygenase-2 expression in DSS-induced UC. As expected, DSS administration to mice for 7 days produced an increase in colonic cyclooxygenase-2 expression (Figure 9(a)) as detected by western blot analysis. The anti-inflammatory effect exerted by shikonin in the intestine was associated with a significant (90%; *P* < 0.01) inhibition of colonic cyclooxygenase-2 expression (Figure 9(a)) in comparison with colitic animals. 

We also examined the effect of shikonin on the activation of NF-*κ*B and pSTAT-3 in the colon homogenates. Transcription factors of the NF-*κ*B family play an essential role in the regulation of genes involved in the immune and inflammatory response. The promoter and enhancing regions of genes coding inflammatory mediators have binding sites for NF-*κ*B [34]. In inflammatory bowel disease, NF-*κ*B activation is observed in inflamed intestinal mucosa, in which it induces the expression of COX-2. Indeed, this activity has shown to be higher in nuclear extracts of *lamina propria* biopsy samples of patients with Crohn's disease and with UC [35] as well as in macrophages and epithelial cells of UC patients [36], suggesting that targeting NF-*κ*B activity in these cells might be an attractive goal for therapeutic intervention [14]. We verified that nuclear p65 levels were markedly higher in mice that received DSS as compared to those that only received normal drinking water (Figure 9(b)). Shikonin partially prevented the translocation of p65 to the nucleus by 57% (*P* < 0.01) (Figure 9(b)) in comparison with the colitic group. Blocking the NF-*κ*B pathway could constitute an interesting approach to break the vicious circle. Specifically, the NF-*κ*B p65 subunit seems to be an important regulator in intestinal inflammation in inflammatory bowel disease; in fact, treatment with antisense or decoy oligonucleotides that inhibit p65 has been shown to ameliorate the macroscopic signs of colitis as well as the nuclear accumulation and DNA binding activity of p65 protein in several models of intestinal inflammation [37–39]. The rectal administration of these antisense oligonucleotides in a model of DSS-induced UC was effective when administered on day 0 or 2 [40, 41]; however, no effect was seen when administered on day 7. These results suggest that treatment with NF-*κ*B antisense oligonucleotides has an inhibitory effect on DSS-induced colitis when administered in the early phases of inflammation. In a previous study using a model of acute ear edema induced by topical 12-*O*-tetradecanoylphorbol 13-acetate application and lipopolysaccharide-stimulated RAW 264.7 macrophages, we demonstrated that shikonin's anti-inflammatory activity occurs through its inhibitory effect on NF-*κ*B activation, which can, in turn, be attributed to its ability to prevent I*κ*B*α* degradation [24]. Moreover, a recent pharmacogenomics study showed that shikonin inhibits a group of genes associated with the early stages of the inflammatory response, including cytokine, chemotaxis, and cell migration genes [42]. Lu et al. [43] recently suggested as a possible biochemical mechanism the inhibition of the proteasome, which consequently inhibits the degradation of I*κ*B*α*, as they demonstrated in lipopolysaccharide-stimulated rat primary macrophages treated with shikonin 1 *μ*M. In the present study, we have demonstrated that shikonin is able to inhibit the expression of COX-2 in colon homogenates of treated mice, probably by preventing the nuclear translocation of the p65 subunit, as was the case with 12-*O*-tetradecanoylphorbol 13-acetate-induced ear edema [24]. 

Recent data indicate that STAT-3 is one of the crucial targets for the treatment of inflammatory bowel disease as the development of colonic inflammation is strongly associated with the induction of STAT-3 activity. Upon activation, this transcription factor translocates to the nucleus [44]. Mudter et al. [45] and Atreya and Neurath [46] found that STAT-3 and pSTAT-3 levels are significantly higher in ulcerative colitis patients in comparison with controls. Its overactivation in monocytes and epithelial cells results in the innate defense system allowing bacteria to fester and eventually initiate the disease [47]. Moreover, STAT-3 and pSTAT-3 levels also correlate with the histologic profile of experimental inflammation tissues [48, 49]; therefore STAT-3 seems to be a potential therapeutic target for ulcerative colitis. In previous work, Bai et al. [50] studied the blockade of STAT-3 by antisense oligonucleotides in trinitrobenzenesulphonic acid-(TNBS-)induced murine colitis. As can be seen in our results, in noncolitic animals, only weak immunoreactivity was detected in the nucleus for pSTAT-3 (Figure 9(c)). Similar results were observed in the group of colitic mice treated with shikonin (55% reduction in comparison with colitic mice, *P* < 0.01). This finding suggests that an important beneficial effect of shikonin in the treatment of inflammatory bowel disease is its ability to reduce the activation of STAT-3. However, more studies are needed to gain further insight into this mechanism.

One aspect that has not been assessed in this work and which may be involved in the protective mechanism of shikonin in experimental ulcerative colitis is the effect the compound may have on the gastrointestinal microbiota, especially since it is administered orally. The intestinal lumen has a large number of microorganisms in perfect symbiosis with the host. These interactions can be beneficial or detrimental. The intestinal immune system must, on the one hand, defend from the attack of pathogens and thus prevent excessive entry of germs through the epithelium and, on the other, recognize the resident intestinal microflora. The breakdown of this balance leads to inflammatory bowel disease. Therefore, an important challenge is to identify and understand these interactions since many factors (diet, medication, therapy with antibiotics, etc.) can modify them. For example, a defect in the permeability of the intestinal mucosa may lead to a loss of tolerance to normal enteric flora. 

The defects in the intestinal epithelial barrier observed in ulcerative colitis lead to an increase in the concentration of anaerobic bacteria. This explains the fact that antibiotics and probiotic agents are more effective and better tolerated than immunosuppressants. The inflammation induced by chemical agents such as DSS produces a profound change in the colonic microbial community (Abraham and Medzhitov, 2011) [51], decreasing the concentration of Bacteroidetes and increasing the relative number of Proteobacteria, including Enterobacteriaceae, so if the host is genetically susceptible, it will respond to such pathogenic flora. Enterobacteriaceae such as *Klebsiella pneumoniae* or *Escherichia coli* have *N*-acetyltransferase activity, which activates certain carcinogens. While shikonin is not antimicrobial against these bacteria, it is able to inhibit the enzyme [52] and therefore play a protective role.

## 4. Conclusion

Shikonin treatment is effective in UC induced by DSS at a dose of 25 mg kg^−1^ (twice *p.o.*). Shikonin reduces proinflammatory cytokine production and inhibits cyclooxygenase-2 and inducible nitric oxide synthase expression in mice with established colitis. The effects of shikonin in this experimental model may be explained not only by an inhibition of Th1 responses, but also by the blockade of the activation of two major targets: NF-*κ*B and STAT-3. This is the first report of shikonin's ability to attenuate the acute inflammation induced by oral administration of DSS. The combination of the known anti-*N*-acetyltransferase activity and wound healing properties of shikonin together with its anti-inflammatory properties make it a promising therapeutic agent for the management of inflammatory bowel disease.

## Figures and Tables

**Table 1:** Scoring system to calculate the disease activity index.

Scoring of disease activity index (DAI**)			
Score	Weight loss	Stool consistency	Visible blood in feces
0	None	Normal	None
1	1–5%		
2	6–10%	Loose	Slight bleeding
3	11–20%		
4	>20%	Diarrhea	Gross bleeding

**The DAI value is calculated as the sum of scores of weight loss, stool consistency, and blood in feces.

**Table 2:** Histologic scoring system for DSS-induced colitis.

Scoring of severity of histological damage		
Feature	Score	Description
Severity of inflammation	0	None
	1	Mild
	2	Moderate
	3	Severe

Extent of inflammation	0	None
	1	Mucosa
	2	Mucosa and submucosa
	3	Transmural

Crypt damage	0	None
	1	1/3 damaged
	2	2/3 damaged
	3	Crypts lost, surface epithelium present
	4	Crypts lost and surface epithelium lost

^∗∗^Scores were calculated by adding the score for the three parameters, giving a maximum score of 10.

## References

[1] Kornbluth A, Sachar DB (2010). Ulcerative colitis practice guidelines in adults: American college of gastroenterology, practice parameters committee. *American Journal of Gastroenterology*.

[2] Stange EF, Travis SPL, Vermeire S (2008). European evidence-based Consensus on the diagnosis and management of ulcerative colitis: definitions and diagnosis. *Journal of Crohn’s and Colitis*.

[3] Ghosh S, Mitchell R (2007). Impact of inflammatory bowel disease on quality of life: results of the European Federation of Crohn’s and Ulcerative Colitis Associations (EFCCA) patient survey. *Journal of Crohn’s and Colitis*.

[4] Lakatos PL (2006). Recent trends in the epidemiology of inflammatory bowel diseases: up or down?. *World Journal of Gastroenterology*.

[5] Bonen DK, Cho JH (2003). The genetics of inflammatory bowel disease. *Gastroenterology*.

[6] Cario E (2008). Barrier-protective function of intestinal epithelial Toll-like receptor 2. *Mucosal Immunology*.

[7] López-Serrano P, Pérez-Calle JL, Pérez-Fernández MT, Fernández-Font JM, Boixeda DM, Fernández-Rodríguez CM (2010). Environmental risk factors in inflammatory bowel diseases. Investigating the hygiene hypothesis: a Spanish case-control study. *Scandinavian Journal of Gastroenterology*.

[8] Bernstein CN, Blanchard JF, Rawsthorne P, Yu N (2001). The prevalence of extraintestinal diseases in inflammatory bowel disease: a population-based study. *American Journal of Gastroenterology*.

[9] Bernstein CN, Wajda A, Blanchard JF (2005). The clustering of other chronic inflammatory diseases in inflammatory bowel disease: a population-based study. *Gastroenterology*.

[10] Lakatos PL, Lakatos L (2008). Risk for colorectal cancer in ulcerative colitis: changes, causes and management strategies. *World Journal of Gastroenterology*.

[11] Sánchez-Muñoz F, Domínguez-López A, Yamamoto-Furusho JK (2008). Role of cytokines in inflammatory bowel disease. *World Journal of Gastroenterology*.

[12] Bouguen G, Chevaux JB, Peyrin-Biroulet L (2011). Recent advances in cytokines: therapeutic implications for inflammatory bowel diseases. *World Journal of Gastroenterology*.

[13] Sánchez-Fidalgo S, Cárdeno A, Villegas I, Talero E, de la Lastra CA (2010). Dietary supplementation of resveratrol attenuates chronic colonic inflammation in mice. *European Journal of Pharmacology*.

[14] Atreya I, Atreya R, Neurath MF (2008). NF-*κ*B in inflammatory bowel disease. *Journal of Internal Medicine*.

[15] Kwon KH, Murakami A, Tanaka T, Ohigashi H (2005). Dietary rutin, but not its aglycone quercetin, ameliorates dextran sulfate sodium-induced experimental colitis in mice: attenuation of pro-inflammatory gene expression. *Biochemical Pharmacology*.

[16] Recio MC, Andújar I, Rios JL (2012). Anti-inflammatory agents from plants: progress and potential. *Current Medicinal Chemistry*.

[17] Maity S, Ukil A, Karmakar S (2003). Thearubigin, the major polyphenol of black tea, ameliorates mucosal injury in trinitrobenzene sulfonic acid-induced colitis. *European Journal of Pharmacology*.

[18] Sugimoto K, Hanai H, Tozawa K (2002). Curcumin prevents and ameliorates trinitrobenzene sulfonic acid-induced colitis in mice. *Gastroenterology*.

[19] Arafa HMM, Hemeida RA, El-Bahrawy AIM, Hamada FMA (2009). Prophylactic role of curcumin in dextran sulfate sodium (DSS)-induced ulcerative colitis murine model. *Food and Chemical Toxicology*.

[20] González R, Ballester I, López-Posadas R (2011). Effects of flavonoids and other polyphenols on inflammation. *Critical Reviews in Food Science and Nutrition*.

[21] Papageorgiou VP, Assimopoulou AN, Couladouros EA, Hepworth D, Nicolaou KC (1999). The chemistry and biology of alkannin, shikonin, and related naphthazarin natural products. *Angewandte Chemie*.

[22] Tan W, Lu J, Huang M (2011). Anti-cancer natural products isolated from chinese medicinal herbs. *Chinese Medicine*.

[23] Zeng YQ (2006). *Identificación y actividad farmacológica de principios de especies antiinflamatorias [Tesis Doctoral]*.

[24] Andújar I, Recio MC, Bacelli T, Giner RM, Ríos JL (2010). Shikonin reduces oedema induced by phorbol ester by interfering with I*κ*B*α* degradation thus inhibiting translocation of NF-*κ*B to the nucleus. *British Journal of Pharmacology*.

[25] Cooper HS, Murthy SNS, Shah RS, Sedergran DJ (1993). Clinicopathologic study of dextran sulfate sodium experimental murine colitis. *Laboratory Investigation*.

[26] Tamaki H, Nakamura H, Nishio A (2006). Human thioredoxin-1 ameliorates experimental murine colitis in association with suppressed macrophage inhibitory factor production. *Gastroenterology*.

[27] Suzuki K, Ota H, Sasagawa S, Sakatani T, Fujikura T (1983). Assay method for myeloperoxidase in human polymorphonuclear leukocytes. *Analytical Biochemistry*.

[28] Siegmund B, Lehr HA, Fantuzzi G, Dinarello CA (2001). IL-1*β*-converting enzyme (caspase-1) in intestinal inflammation. *Proceedings of the National Academy of Sciences of the United States of America*.

[29] Grisham MB, Johnson GG, Lancaster JR (1996). Quantitation of nitrate and nitrite in extracellular fluids. *Methods in Enzymology*.

[30] Jurjus AR, Khoury NN, Reimund JM (2004). Animal models of inflammatory bowel disease. *Journal of Pharmacological and Toxicological Methods*.

[31] Papadakis KA, Targan SR (2000). Role of cytokines in the pathogenesis of inflammatory bowel disease. *Annual Review of Medicine*.

[32] Dai Q, Fang J, Zhang FS (2009). Dual role of shikonin in early and late stages of collagen type II arthritis. *Molecular Biology Reports*.

[33] Alexander JS, Chaitanya GV, Grisham MB, Boktor M (2010). Emerging roles of lymphatics in inflammatory bowel disease. *Annals of the New York Academy of Sciences*.

[34] Ghosh S, Hayden MS (2008). New regulators of NF-*κ*B in inflammation. *Nature Reviews Immunology*.

[35] Schreiber S, Nikolaus S, Hampe J (1998). Activation of nuclear factor *κ*B inflammatory bowel disease. *Gut*.

[36] Rogler G, Brand K, Vogl D (1998). Nuclear factor *κ*B is activated in macrophages and epithelial cells of inflamed intestinal mucosa. *Gastroenterology*.

[37] Neurath MF, Pettersson S, Meyer Zum Büschenfelde KH, Strober W (1996). Local administration of antisense phosphorothioate oligonucleotides to the p65 subunit of NF-*κ*B abrogates established experimental colitis in mice. *Nature Medicine*.

[38] Spiik AK, Ridderstad A, Axelsson LG, Midtvedt T, Björk L, Pettersson S (2002). Abrogated lymphocyte infiltration and lowered CD14 in dextran sulfate induced colitis in mice treated with p65 antisense oligonucleotides. *International Journal of Colorectal Disease*.

[39] Fichtner-Feigl S, Fuss IJ, Preise JC, Strober W, Kitani A (2005). Treatment of murine Th1- and Th2-mediated inflammatory bowel disease with NF-*κ*B decoy oligonucleotides. *Journal of Clinical Investigation*.

[40] Xiang JY, Wu LG, Huang XL (2009). Amelioration of murine dextran sulfate sodium-induced colitis by nuclear factor-*κ*b decoy oligonucleotides. *American Journal of Surgery*.

[41] Murano M, Maemura K, Hirata I (2000). Therapeutic effect of intracolonically administered nuclear factor *κ*B (p65) antisense oligonucleotide on mouse dextran sulphate sodium (DSS)- induced colitis. *Clinical and Experimental Immunology*.

[42] Chiu SC, Tsao SW, Hwang PI, Vanisree S, Chen YA, Yang NS (2010). Differential functional genomic effects of anti-inflammatory phytocompounds on immune signaling. *BMC Genomics*.

[43] Lu L, Qin A, Huang H (2011). Shikonin extracted from medicinal Chinese herbs exerts anti-inflammatory effect via proteasome inhibition. *European Journal of Pharmacology*.

[44] Fan Y, Zhang YL, Wu Y (2008). Inhibition of signal transducer and activator of transcription 3 expression by RNA interference suppresses invasion through inducing anoikis in human colon cancer cells. *World Journal of Gastroenterology*.

[45] Mudter J, Weigmann B, Bartsch B (2005). Activation pattern of signal transducers and activators of transcription (STAT) factors in inflammatory bowel diseases. *American Journal of Gastroenterology*.

[46] Atreya R, Neurath MF (2008). Signaling molecules: the pathogenic role of the IL-6/STAT-3 trans signaling pathway in intestinal inflammation and in colonic cancer. *Current Drug Targets*.

[47] Li Y, de Haar C, Peppelenbosch MP, van der Woude CJ (2012). New insights into the role of STAT3 in IBD. *Inflammatory Bowel Disease*.

[48] Lovato P, Brender C, Agnholt J (2003). Constitutive STAT3 activation in intestinal T cells from patients with Crohn’s disease. *Journal of Biological Chemistry*.

[49] Suzuki K, Sugimura K, Hasegawa K (2001). Activated platelets in ulcerative colitis enhance the production of reactive oxygen species by polymorphonuclear leukocytes. *Scandinavian Journal of Gastroenterology*.

[50] Bai AP, Hu PJ, Chen J (2007). Blockade of STAT3 by antisense oligonucleotide in TNBS-induced murine colitis. *International Journal of Colorectal Disease*.

[51] Abraham C, Medzhitov R (2011). Interactions between the host innate immune system and microbes in inflammatory bowel disease. *Gastroenterology*.

[52] Kuo HM, Hsia TC, Chuang YC, Lu HF, Lin SY, Chung JG (2004). Shikonin inhibits the growth and N-acetylation of 2-aminofluorene in *Helicobacter pylori* from ulcer patients. *Anticancer Research*.

